# Umbilical Cord Blood Mesenchymal Stem Cells as an Infertility Treatment for Chemotherapy Induced Premature Ovarian Insufficiency

**DOI:** 10.3390/biomedicines7010007

**Published:** 2019-01-18

**Authors:** Sara A. Mohamed, Shahinaz Shalaby, Soumia Brakta, Lelyand Elam, Amro Elsharoud, Ayman Al-Hendy

**Affiliations:** 1 Division of Translation Research, Department of Obstetrics and Gynecology, Medical College of Georgia Augusta University, Augusta, GA 30912, USA; Sarro_2010@hotmail.co.uk (S.A.M.); shahinazmshams@yahoo.com (S.S.); sbrakta@augusta.edu (S.B.); LSTONE4@Lsu.edu (L.E.); 2 Department of Obstetrics and Gynecology, Mansoura Faculty of Medicine, Mansoura University Hospital, Mansoura 35511, Egypt; 3 Department of Pharmacology, Tanta Faculty of Medicine, Tanta 31511, Egypt; 4 Department of Obstetrics and Gynecology, University of Illinois, Chicago, IL 60612, USA; amr.elsharoud@gmail.com

**Keywords:** ovarian failure, chemotherapy, stem cells

## Abstract

Background: Premature ovarian insufficiency (POI) is a challenging disease, with limited treatment options at the moment. Umbilical cord blood mesenchymal stem cells (UCMSCs) have demonstrated promising regenerative abilities in several diseases including POI. Materials and Method: A pre-clinical murine case versus vehicle control randomized study. Two experiments ran in parallel in each of the three groups. The first was to prove the ability of UCMSCs in restoring ovarian functions. The second was to prove improved fertility. A total of 36 mice were randomly assigned; 6 mice into each of 3 groups for two experiments. Group 1 (control), group 2 (sham chemotherapy), group 3 (stem cells). Results: In the first experiment, post-UCMSCs treatment (group 3) showed signs of restored ovarian function in the form of increased ovarian weight and estrogen-dependent organs (liver, uterus), increased follicular number, and a significant decrease in FSH serum levels (*p* < 0.05) compared to group 2, and anti-Mullerian hormone (AMH) serum levels increased (*p* < 0.05) in group 3 versus group 2. Immuno-histochemistry analysis demonstrated a higher expression of AMH, follicle stimulating hormone receptor (FSHR) and Inhibin A in the growing follicles of group 3 versus group 2. In the second experiment, post-UCMSCs treatment (group 3) pregnancy rates were higher than group 2, however, they were still lower than group 1. Conclusion: We demonstrated the ability of UCMSCs to restore fertility in female cancer survivors with POI and as another source of stem cells with therapeutic potentials.

## 1. Introduction

Premature ovarian failure (POF) recently known as premature ovarian insufficiency (POI) is a disease with a challenging nature and multiple complexities [1]. POF is classified by the World Health Organization (WHO) as hypergonadotrophic hypogonadism [2]. In other words, menopausal levels of follicle stimulating hormone (FSH > 40 IU/L) and low estradiol levels, assessed in two separate settings at least four weeks apart besides amenorrhea from 4–6 months in women under age of 40 [3].

In 70% of POI cases, it is unlikely that a single specific cause can be identified. Although, many factors have been reported, including genetic, autoimmune, or prior anti-cancer treatment, either surgical, radiotherapy, or chemotherapy; in many cases, the cause remain unknown [4].

POI has been shown to be associated with a loss of both ovulation and hormonal secretory functions. Anovulation certainly leads to infertility. The incidence of other medical conditions also increases as a result of decreased ovarian estrogen secretion, including Alzheimer’s, cardiovascular, autoimmune diseases, metabolic syndrome, osteoporosis, diabetes, and gynecological cancers [5]. 

The mechanism of ovarian failure is most likely accelerated by follicular atresia, but the detailed pathogenesis is yet to be fully understood [6]. In many cases, resting primordial follicles remain visible in these ovaries, but they fail to respond to the abundantly available FSH and luteinizing hormone (LH) [3].

No therapeutic intervention has been proven to be effective in restoring fertility in patients with POI, especially with hormonal replacement therapy (HRT), which has been used clinically despite its noticeable side effects and the fact that, it neither suppresses the FSH rise, nor mends the impaired ovarian function [7]. Currently, ovum donation remains the only reliable method to establish pregnancy in women with POI. Even though this approach is attainable, offspring will not be genetically related to the recipient mother. Furthermore, ova donation is not ethically acceptable for many couples due to various cultural and religious reasons [8]. 

Multiple attempts on ovarian stimulation are usually unsuccessful. That explains why the diagnosis of POI leads to severe physical and emotional suffering among patients. There is a serious need to develop novel effective advances for the treatment of POF [9].

The evidence suggests that bone marrow-derived mesenchymal stem cells (BMSCs) can restore the structure and function of injured tissues [3,9]. During embryologic development, cells of the mesodermal layer can develop to different mesenchymal tissue, including bone, cartilage, tendon, muscle, fat, and marrow stroma [2]. These precursor cells are also present in the post-natal organism as mesenchymal stem cells (MSCs). These stem cells are able to retain their developmental potential after extensive sub-cultivation in vitro [9]. Implantation of culture-expanded mesenchymal stem cells have been confirmed to effect tissue regeneration in multiple animal models. These cells depend on local factors to stimulate their differentiation into the appropriate phenotype [8].

Cell therapy, especially of mesenchymal stem cells, has documented positive outcomes in POI. Different sources of mesenchymal stem cells have been used in the treatment of POI animal models. Moreover, clinical trials are attempting to correlate the MSCs to the resumption of the ovarian activity [9]. We have recently reported that intra-ovarian administered BMSCs are able to restore ovarian hormone production and to reactivate folliculogenesis in the chemotherapy-induced ovarian failure mouse model [10].

The human umbilical cord was successfully tested as a source of MSCs for cell therapy [2]. These MSCs can differentiate into many others cells of the same mesodermal origin but more importantly, they elicit no immune response, owing to the low expression of human leukocyte antigen (HLA), MHC Class I (major histocompatibility antigen), and absence of MHC class II [11]. 

Herein in this study, we used mesenchymal stem cells extracted from cord blood as an abundant source of accessible, non-immunogenic stem cells with noninvasive techniques. The umbilical cord blood mesenchymal stem cells were tested in this preclinical model to prove the concept of using them as an alternative therapeutic option for chemotherapy induced POI.

## 2. Materials and Methods

### 2.1. Animals 

Strain: C57BL6 mice Weight: 20–25 g Age: 4–6 weeks.

Company: Charles River Co (Wilmington, Massachusetts, MA, USA)

Housing: in groups (total numbers; *n* = 36 female mice for both experiments and *n* = 3 male mice for the breeding) in polyethylene cages and kept in the animal facility for one week before enrollment in the study. 

Environment: light/dark cycles 12 h each, controlled room temperature at 22 °C and humidity 50–60%, free water and chewable mouse pellets purchased commercially. 

All animal procedures were performed in accordance with the National Research Council Guide for Care and Use of Laboratory Animals with the approval of Augusta University Institutional Animal Care and Use Committee (IACUC: 2014-0633 15 April 2014). Approval of human cell use was granted by Augusta University Institutional Review Board (IRB 1 June 2014).

### 2.2. Animal Procedures

Two parallel experiments were conducted in this study: (1) treatment experiment, and (2) breeding experiment.

Surgical procedures were done under strict aseptic conditions with general anesthesia (isoflurane) then the fascia and skin were closed using vicryl 3 (ETHICON USA) zero sutures. The recovery post-operative was under a red lamp to keep the animals warm.

### 2.3. The Treatment Experiment

**Figure 1:**
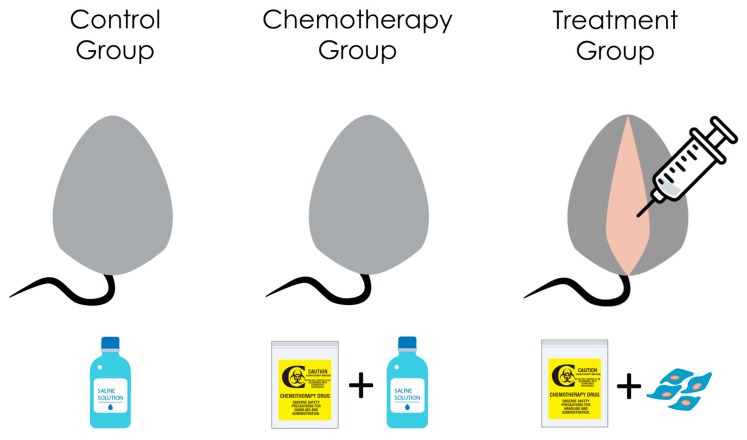
Illustrates different experiment groups: (1) Control group, no chemotherapy is given, (2) chemotherapy group, and no stem cell therapy, only saline was injected, (3) treatment group, the test group, where stem cells were injected in both ovaries.

Accustomed mice were allotted randomly to three different groups, and each had 6 mice; group 1; the control group (no chemotherapy, surgery done to inject saline as placebo), group 2; the chemotherapy group with sham stem cell treatment (chemotherapy was given and the surgery done to inject saline), group 3; the stem cell-based treatment after chemotherapy (test group). A combination chemotherapy (CTX) of busulfan (12 mg/kg) and cyclophosphamide (70 mg/kg) (Sigma Aldrich., St. Louis, MO, USA) administered intra-peritoneal to mice in groups 2 and 3. All groups have been subjected to laparotomy, but with different injection material in each; group 1 and 2 injected with 10 mL phosphate buffered saline/ovary, but in group 3 the injection was UCMSCs (Umbilical cord Mesenchymal Stem cells) (purchased from HemoGenix Inc., Colorado Springs, CO, USA)(catalogue number C-MSC-F1), with the following cluster designation antigens (Phenotype) (according to the supplier): CD45+, D7FIB+, CD44+, CD54+, CD73+, CD90+, CD105+, CD140b+, CD166+, CD146+, HLA-DR-, CD14-, CD19-, CD31-, CD34-, CD41a-, CD235a-, ALP-, CD271+. Cell viability in-vitro was confirmed by plating in culture, then recollecting and counting according to the manufacturer’s instructions. UCMSCs, suspended in 10 mL of phosphate-buffered saline, were injected with 10-uL G30 Hamilton syringe (Harvard Apparatus, Massachusetts, MA, USA) into both ovaries, at a concentration of 5 × 10^5^ cells per ovary, seven days post-chemotherapy (Figure 1). 

### 2.4. The Breeding Experiment

This experiment considered as a duplicate of the treatment experiment. Mice were distributed randomly into the same 3 groups (*n* = 6 each) but with the extension for breeding of with weight and age-matched male mice. This cohabitation designed after Harem theory; 2 female mice: 1 male mice in the same cage starting one week after surgery. The resulting pups were examined carefully for any abnormality and counted per animal in each group. 

### 2.5. Experiment Timeline

In the treatment experiment, we set 4 fixed time points for the blood sample collection and planned different analysis techniques. Time points were 2, 4, 6, and 8 weeks after surgery. Each mouse was weighed and subjected to a 200 µL retro-orbital blood sample collection for hormonal level detection (Estradiol, anti-Mullerian hormone (AMH), and FSH) by the University of Virginia’s Center for Research in Reproduction Ligand Assay and Analysis Core, E2 was measured in the mouse’s serum by an enzyme-linked immunosorbent assay (ELISA; Rodent Estradiol ELISA; CalBiotech, Spring Valley, CA, USA). Assay precision was 6.1% (intra-assay) and 8.9% (inter-assay). Functional sensitivity was 3.0 pg/mL. Radio immune assay (RIA) was used to detect FSH using the reagents provided by the National Hormone and Peptide Program. Assay precision was 6.9% (intra-assay) and 9.4% (inter-assay). Functional sensitivity was 3.0 ng/mL. AMH was measured using a commercial ELISA kit (Rat/Mouse AMH ELISA; Ansh Labs, Webster, TX, USA). Assay precision was 3.6% (intra-assay) and 8.5% (inter-assay). Functional sensitivity was 0.28 ng/mL.

Only one mouse from each group was sacrificed (CO_2_ asphyxiation in accordance with Augusta University animal facility protocols), ovarian tissues were excised and fixed then sent to the histology core where hematoxylin and eosin (H&E) was done to assess the distribution of ovarian follicular development and immunohistochemistry (IHC) staining to assess ovarian expression of follicular-stimulating hormone receptor (FSHR), inhibin A and AMH. Other animals’ organs were also dissected and fixed when further studies were needed. All tissues were handled according to the in-house core facilities protocols as described in our previous experiment [10]. The list of antibodies used for immune-histochemistry: 1-Rabbit anti-human AMH (MIS)(H-300) (Santa Cruz, CA, USA) IF (1:200) Catalogue number SC28912, 2-Rabbit anti-human FSHR (H-190) IF(1:200) Catalogue number SC13935, 3-Rabbit anti-human Inhibin A (H-134) IF(1:200) catalogue number SC-50288, 4-Rabbit anti-human Inhibin B (H-120)IF(1:200) Catalogue number SC-30146. Blocking solution, that was purchased from Beyotime Institute of Biotechnology, and the tissues were incubated with the primary antibodies at 4 °C overnight. Visualization of FSHR-positive cells, and avidin-biotin- peroxidase system with diaminobenzidine as the chromogen (EnVision+ System-HRP (DAB) Code K4011 DakoCytomation, Glostrup, Denmark), was used according to the manufacturer’s instruction. Sections were washed in distillated water and counter-stained with hematoxylin. For negative control, specimens were processed in the absence of the primary antibody. Positive staining was defined microscopically by visually identifying brown pigmentation. The histology slides were assessed in the imaging core with an inverted microscope Icore Axioplane 2 Nikon TE2000-E. The threshold was kept constant for all slides and the background subtraction was set from the region of interest (ROI). A semi-quantitative analysis of the mean intensities of all slides in the three groups was performed [12].

At the end of this section, the study encompassed two parallel experiments; each hormonal profiles were assessed, by measuring hormonal levels in the serum and the display hormonal change effects on the tissues through H&E and IHC staining.

### 2.6. Statistical Analysis

SAS 9.4 (SAS Institute Inc, Cary, NC, USA) was used. The overall significance level was set at 0.05 Bonferroni method to adjust for multiple comparisons where appropriate.

## 3. Results 

### 3.1. UCMSCs Effect on Body Weight

**Figure 2:**
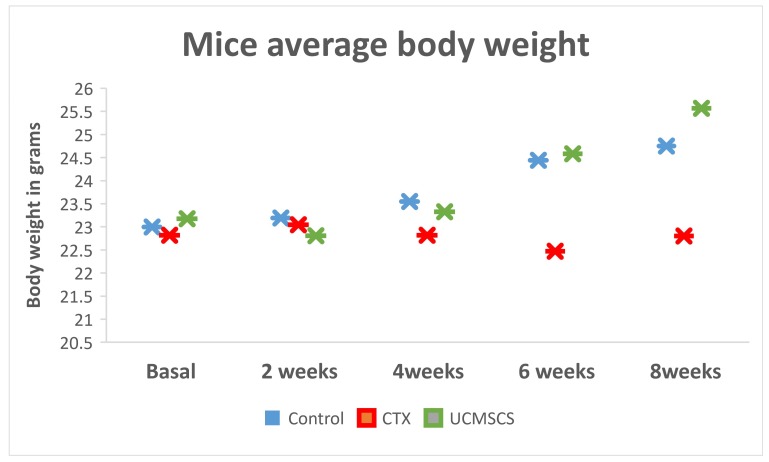
The diagram is showing the trend for increase in body weight in the stem cells group in comparison to significant decrease in the combination chemotherapy (CTX) group.

Total body weight of the mice in group 3 increased substantially, together with ovaries and other estrogen-dependent organs (uterus and liver) two weeks after stem cells implantation (Figure 2).

### 3.2. UCMSCs Recover Ovarian Secretory Functions

**Figure 3:**
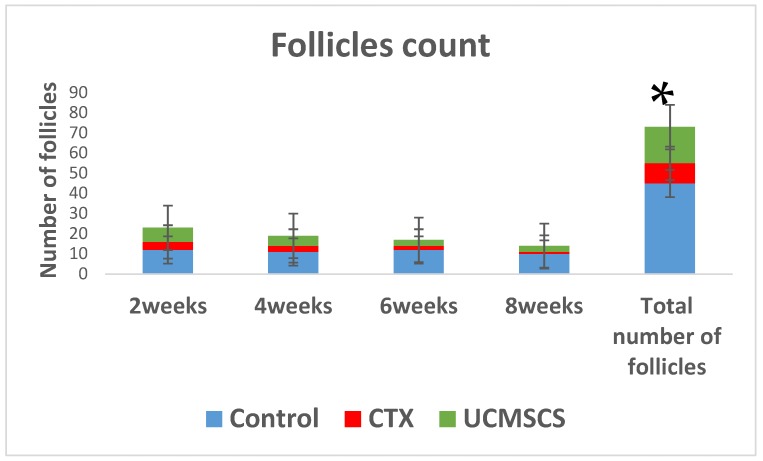
The follicles numbers are illustrated as percentage bars for the experiment groups, which showed the natural decline in control group in blue and the significant decline in CTX group in red, however, in total, the follicle count in umbilical cord blood mesenchymal stem cells (UCMSCs) group in green was significantly increased in the CTX group (* *p* < 0.05).

Comparing number of follicles in total between groups came out in favor for group 3 with a *p* value <0.05, two weeks from the time of stem cells implantation (Figure 3).

**Figure 4:**
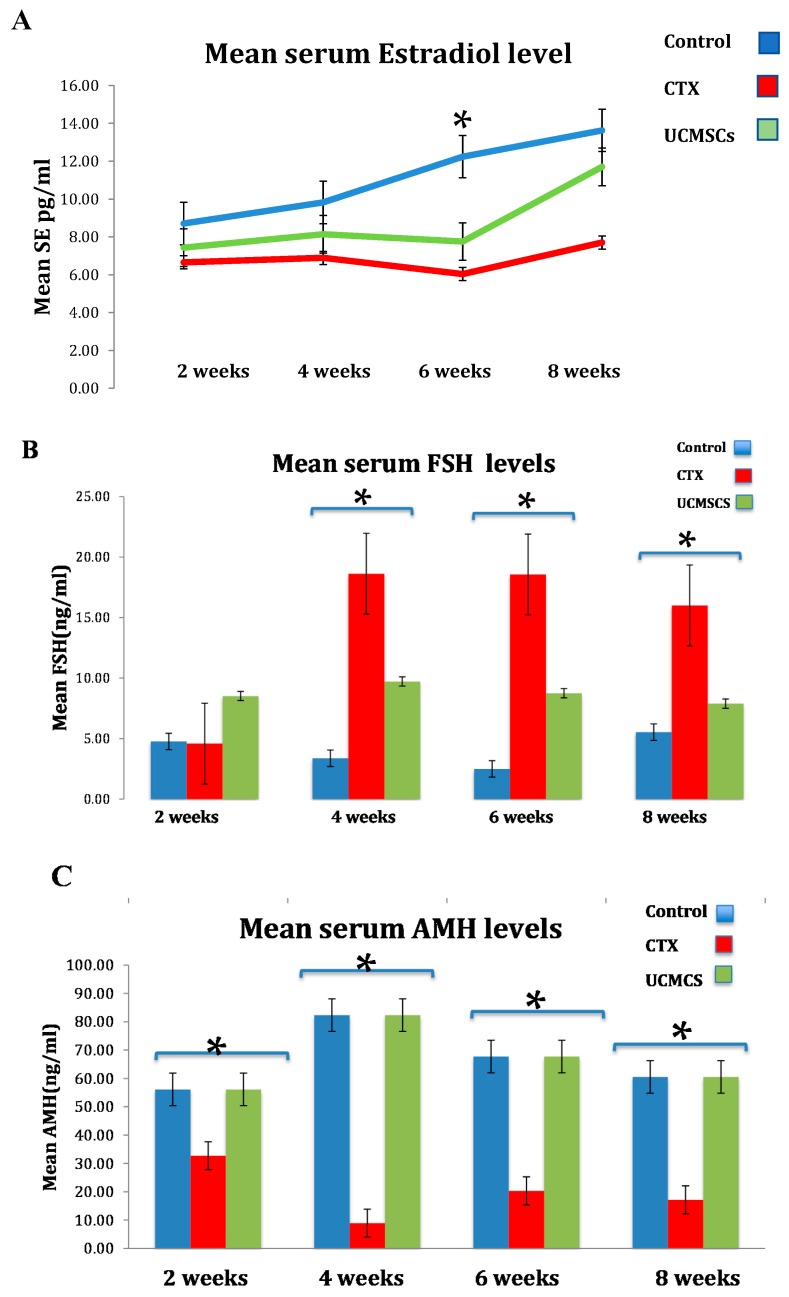
UCMSCs significantly recover ovarian hormonal profile as illustrated by the bar graph in (**A**) Estradiol levels explained the rise in favor of UCMSCs; (**B**) Low follicle stimulating hormone (FSH) levels were significantly represented in UCMSCS from 4 weeks and over; (**C**) anti-Mullerian hormone (AMH) levels were significantly increased in UCMSCS groups in all time points (* *p* < 0.05).

The hormonal profile for group 3 in contrast to group 2 was displayed as a significant decrease of serum FSH levels by 40% ± 5 (*p* < 0.05) and a noteworthy increase by 50% ± 2 (*p* < 0.05) in serum AMH levels. Moreover, Estradiol declined significantly in the CTX group than the control, however, the UCMSCs group exposed an escalating attitude towards normal levels in the control group (Figure 4).

**Figure 5:**
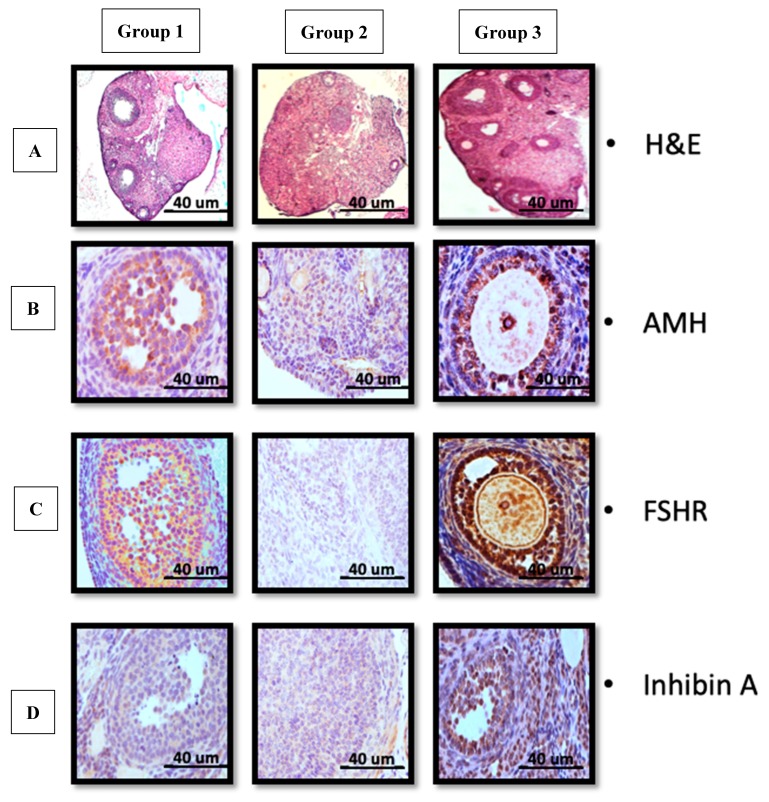
UCMSCs recover ovarian secretory function as shown in (**A**) H&E for group 1 (control) average follicles number and normal tissue distribution, group 2 (CTX) no follicles could be determined and atrophied ovarian tissue, group 3 (UCMSCS) increased number of follicles with revived ovarian tissue. (**B**–**D**) group 1 and 3 confirmed increased expression of AMH and follicular-stimulating hormone receptor (FSHR) and Inhibin A in contrast to group 2 respectively. (Scale bar 40 µm)

Immunohistochemistry analysis validated higher expression of AMH, FSHR and Inhibin A in growing follicles in group 3 versus group 2 (Figure 5).

### 3.3. UCMSCs Rescue Ovarian Excretory Function (Ovulation)

**Figure 6:**
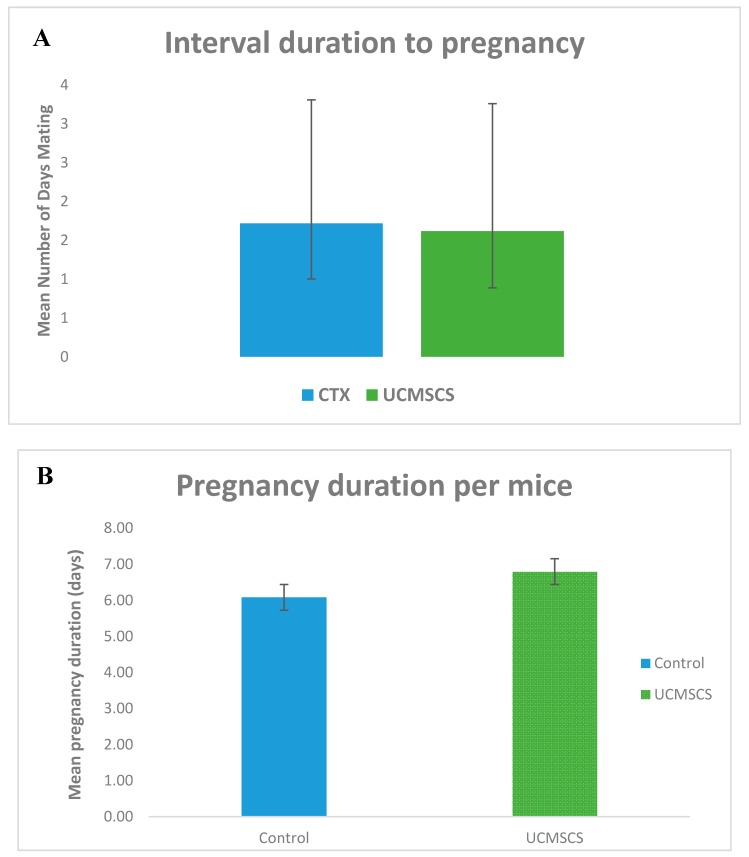

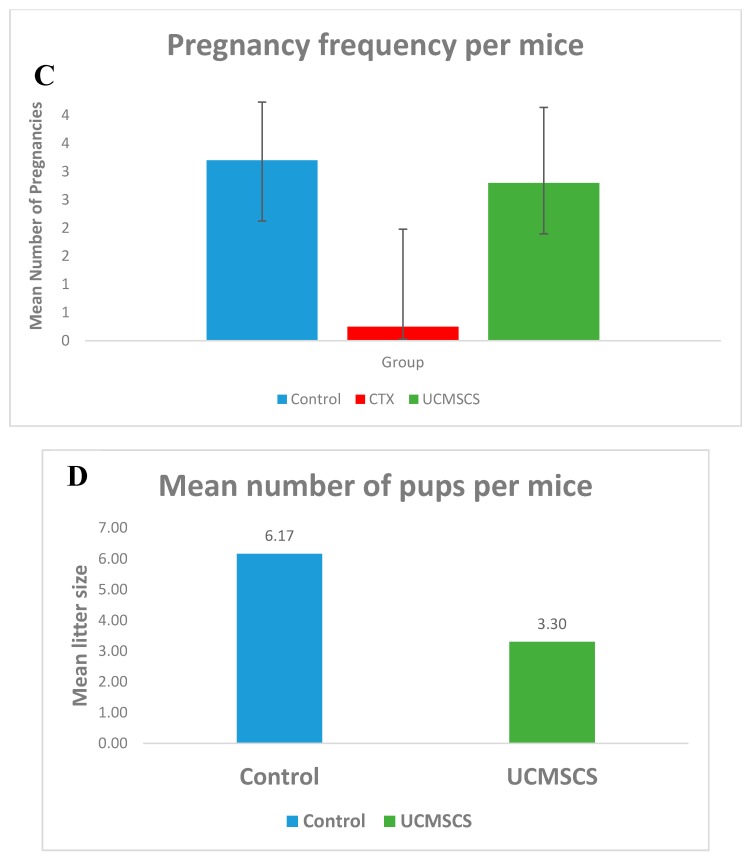
Breeding experiment results, (**A**) and (**B**) UCMSCS group showed more days until pregnancy occur, and slight prolonged pregnancy duration, respectively, however, not statistically significant, (**C**) and (**D**) UCMSCS group exhibited statistical significant data *p* < 0.05 for both, the number of times mice become pregnant, and how many pups were produced, in order.

**Figure 7:**
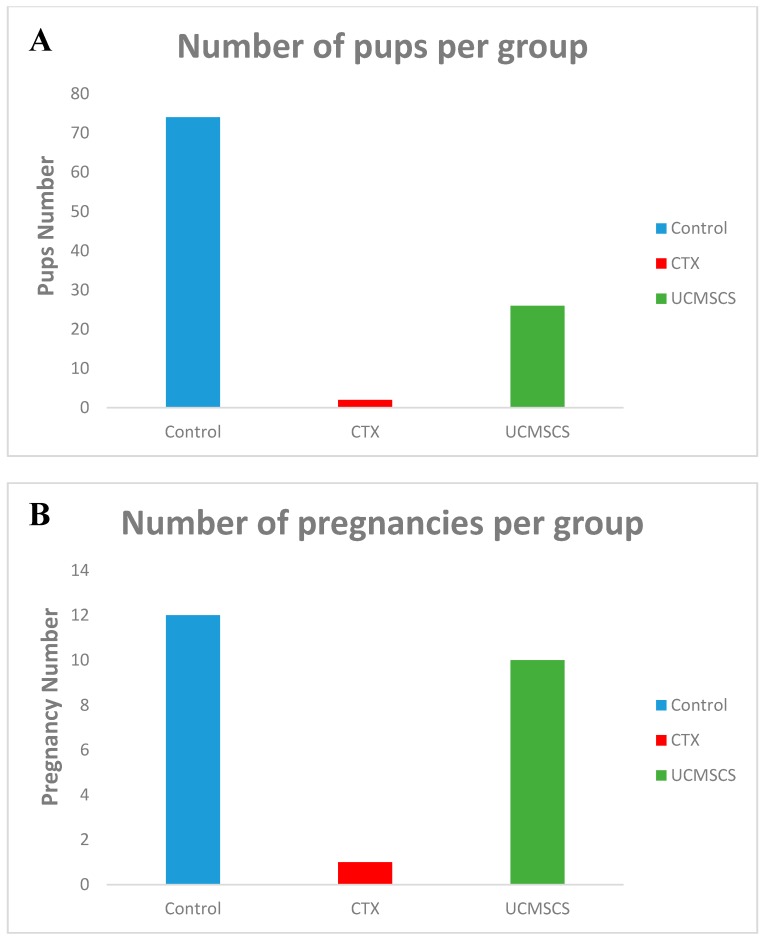
(**A**) Increase in number pf pups in UCMSCS group although not to the range of the control group; (**B**) The pregnancy frequency was in comparable to the control group.

During the three months of breeding, the mating rate was approximately 100% in all group 1 and 3 mice in contrast to group 1 the mating rate was only 25%. Moreover, the number of pups produced were 26 in total for group 3 but only two pups resulted from one pregnancy in group 2. Notably, the latency period (days to get pregnant) showed no significant difference between groups. Crucially, mating levels were evidently higher in group 3 than group 2 but to lesser extent lower that group 1. Scale bar 40 μm. (Figure 6 and Figure 7). Considering general physical condition, all mice in group 3 had normal appearance, but there were two pups in group 2 experienced total body weight loss. 

## 4. Discussion

The key control of folliculogenesis is the endocrine system. Whenever disrupted; the process of follicular development and storage will be disordered resulting in the ovarian pathology, known as POI [13,14].

Cellular therapy: Stem cells have become the central constituent of regenerative medicine [15]. The unique properties of the MSCs make them ideal for cell-based therapy. They have multi-lineage potential and the ability to differentiate into various cell types [1]. They also exhibit promotion in tissue repair by their anti-apoptotic, cytoprotective effects, and angiogenic capacity. Bone marrow mesenchymal stem cells have been linked to success in the reversal of chemotherapy ovarian damage, but its underlying mechanism has not yet been revealed [10,16].

Previous studies have shown that UCMSCs presumably function through the paracrine pathway. In other words, they enhance the environmental factors surrounding the ovarian milieu to better function without differentiating into any of the follicular elements, neither oocytes or granulosa cells due to the absence of evaluating evidence of any meiotic marker to confirm that these cells are haploid [17,18].

Some reports suggest that therapeutic activity of the human placental mesenchymal stem cell (hMSCs) could be related to the secretion of collagen, and their effects have been attributed to the fact that they exhibit higher telomerase activity, more pluripotent markers expression, a higher expression of transcription factor-like OCT4, and NANOG, which can improve the production of cytokines via the JAK/STAT pathway [19,20]. Although hUCMSCs (human Umbilical cord mesenchymal stem cells) have the ability to differentiate into germ cells, recent theories and reports tend to deem that they do not actually change in vivo [19]. 

The reversibility of the chemotherapy damage depends on patient age, exposure degree, and ovarian reserve at the time of exposure [21,22]. 

UCMSCs are considered as an infinite source of stem cells make them an interesting source of stem cells and regenerative medicine. They can be isolated from human umbilical cords in large amounts, expanded in cultures, frozen/thawed, and engineered [23,24].

In this study, we have found that after administering UCMSCs, both functions of the ovary were restored, that is; (1) the endocrine functions of estrogen production in the form of weight gain and increase in weight of estrogen-dependent organs like ovaries, and (2) exocrine function of oocytes that result in successful pregnancies and delivery of healthy pups. These results were achieved by many other researchers [2,4,21,25]. 

Our experiments showed the effect of UCMSCs on the chemotherapy damaged ovaries in mice; herein we did not elaborate on the detailed mechanisms, which we are continuing to study in the current running research.

The present study used a certain combination of chemotherapy as a result of the two experiments (data not represented), which target the therapeutic dose to affect ovarian damage without loss of the animals. Furthermore, the testing started one week after the injection of the cells and two weeks from chemotherapy, taking into consideration the right hit of the therapeutic window, mentioned in earlier research, stated a decline in ovarian function with time after chemotherapy treatment [25].

Additionally, UCMSCs manifested a long-time survival in ovarian tissues with no verification of its proliferation. This provides great insight for human use [25]. It is suggested that the mechanism could be through the inhibition of granulosa cell apoptosis and follicular atresia by the upregulation of AMH and FSHR expression of granulosa cells [6]. While this study did not target the molecular signaling or extend the time required to evaluate such concern, we hold the same opinion with other researchers for that concept.

We considered that the successful engraftment of the cells without any signs of immune rejection is a proof of the hypothesis that these cells are a robust source of stem cells. More importantly, the xenotransplantation, human to animal, that yielded physically normal pups without any hybrid model is another proof that these cells are not differentiating. They improve the micro-environment and hence counteract any idea of genetic mixing [26].

The pivotal concern in applying stem cells in practice is adjusting the dose and method of administration in order to optimize outcomes. Although there are still various debates, all the foreseen results are very promising. The transplantation approaches differ between researchers [2] in favor of the intravenous injections through tail vein (which will pave the way for easier human application), and the boomerang effect of repairing other damaged organs, other than ovaries. Our group, however, did not find this rationale plausible because of the thrombus formation risk, as cells are large in size or not adequately dissociated.

Although recent reports have mentioned the use of hPMSC transplantation in the restoration of ovarian function, there were no significant differences in the results between different origins when studied on wound healing. There was a significant difference when MSCs derived from the three different components of the umbilical cord were differentiated into osteoblasts, adipocytes, and chondrocytes, with varying precursor potentials. These results will promote an interest to study the effect of each cell type in treating POI comparatively, so that the most promising cell type can be determined in future work [27].

This work demonstrated that UCMSCs possess a sketchier display to improve the functionality of ovaries damaged by chemotherapy. With the incremental increase of females who lose their fertility due to different underlying reasons, including due to age, genetic, chemotherapy treatment, or spontaneously; the UCMSCs should be considered a worthwhile source of stem cells that can rejuvenate fertility potential.
